# Comprehensive Oral Rehabilitation With Corticobasal Implants: Restoring Function and Aesthetics

**DOI:** 10.7759/cureus.114012

**Published:** 2026-08-05

**Authors:** Ruby Singh, Mayank Singh, Lakshya Kumar, Pooran Chand

**Affiliations:** 1 Prosthodontics, King George's Medical University, Lucknow, IND

**Keywords:** corticobasal implants, flapless surgery, full mouth rehabilitation, graftless implantology, immediate loading, multi-unit abutment, two-piece system

## Abstract

This case report describes the comprehensive oral rehabilitation of a 35-year-old male patient using corticobasal implants. The patient presented with multiple missing teeth, immobile remaining teeth, and compromised residual alveolar bone. Due to limited bone availability and the patient’s desire for a fixed solution without grafting, a corticobasal implant system was selected. Implants were placed using an immediate-loading protocol that engaged the cortical bone to achieve primary stability. The final prosthesis restored both aesthetics and function, with significant improvement in masticatory efficiency and patient satisfaction. Corticobasal implantology provided a predictable, minimally invasive, and graftless alternative for full-mouth rehabilitation in compromised bone conditions.

## Introduction

Rehabilitation of patients with severely resorbed alveolar ridges remains a significant challenge in implant dentistry because inadequate bone volume often limits the placement of conventional endosseous implants and may necessitate extensive bone augmentation procedures. Corticobasal implantology has emerged as an alternative treatment modality for such patients by utilizing the highly mineralized basal cortical bone rather than relying predominantly on the cancellous alveolar bone for primary stability. Unlike conventional crestal implants, corticobasal implants achieve bicortical anchorage in dense cortical bone, which may allow implant placement in compromised ridges while reducing or eliminating the need for procedures such as bone grafting or sinus augmentation in appropriately selected patients [1,2].

One of the distinguishing features of corticobasal implants is their ability to support immediate functional loading, often within 48-72 hours after placement, provided adequate primary stability is achieved [3]. This approach may shorten the overall treatment duration and reduce the period during which patients remain edentulous. Cortical bone engagement provides high primary stability, allowing their use in selected patients with reduced bone volume or compromised bone quality when appropriate clinical protocols are followed [4].

From a biomechanical perspective, corticobasal implants transfer occlusal forces to the dense cortical bone, which may contribute to favorable load distribution and functional stability [5]. In appropriately selected cases, the single-stage, flapless, and graftless treatment approach may reduce surgical morbidity, shorten rehabilitation time, and lower overall treatment costs compared with conventional implant protocols requiring augmentation procedures [6]. Although encouraging clinical outcomes have been reported, the long-term evidence supporting corticobasal implantology remains limited compared with that for conventional implant systems, and successful treatment is highly dependent on careful case selection, meticulous surgical execution, and prosthetic planning.

This case report aims to describe the comprehensive oral rehabilitation of a 35-year-old male patient with generalized horizontal bone loss, reduced posterior alveolar height, and compromised dentition who desired a fixed prosthetic solution without bone augmentation. These clinical findings made corticobasal implant therapy an appropriate treatment option. Although several studies have reported favorable outcomes with corticobasal implants, detailed clinical reports describing comprehensive full-mouth rehabilitation using immediate-loading protocols in patients with generalized horizontal bone loss remain relatively limited. Additional well-documented case reports may therefore contribute to the growing body of clinical evidence.

## Case presentation

**Figure 1:**
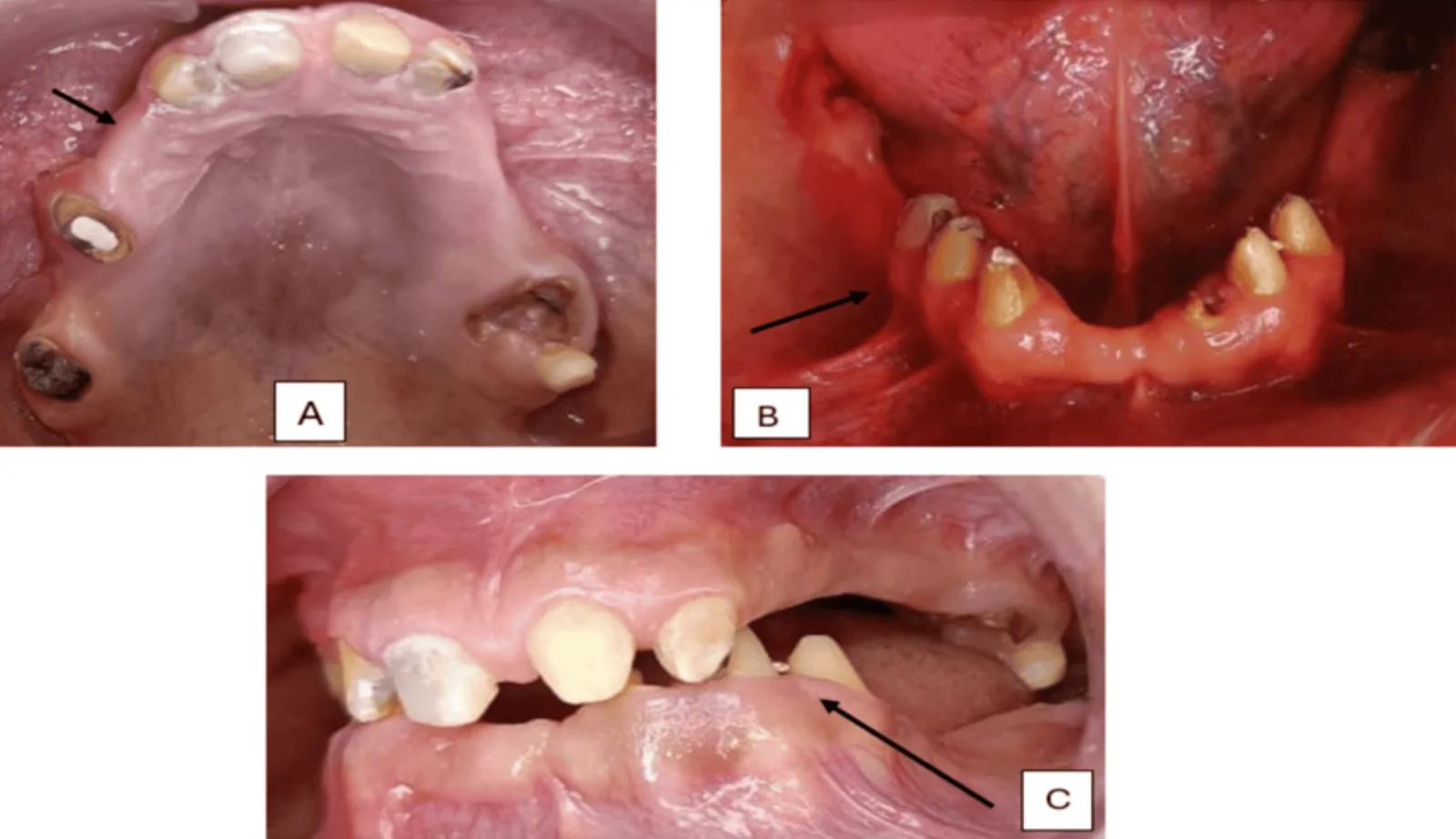
Preoperative intraoral photographs of the patient. (A) Maxillary arch (black arrow indicates the resorbed ridge). (B) Mandibular arch (black arrow shows multiple missing posterior teeth and a collapsed occlusal surface). (C) Maximum intercuspation view demonstrating severe loss of the vertical dimension of occlusion (black arrow).

A 35-year-old male presented to the Department of Prosthodontics with a chief complaint of difficulty chewing and dissatisfaction with his appearance due to multiple missing posterior teeth in both the maxilla and mandible (Figure 1A-1C). The patient reported a medical history of low calcium levels and a dental history of brittle teeth that fractured spontaneously, as well as failed fixed partial denture (FPD) treatment in the maxilla and mandible one year earlier.

**Figure 2:**
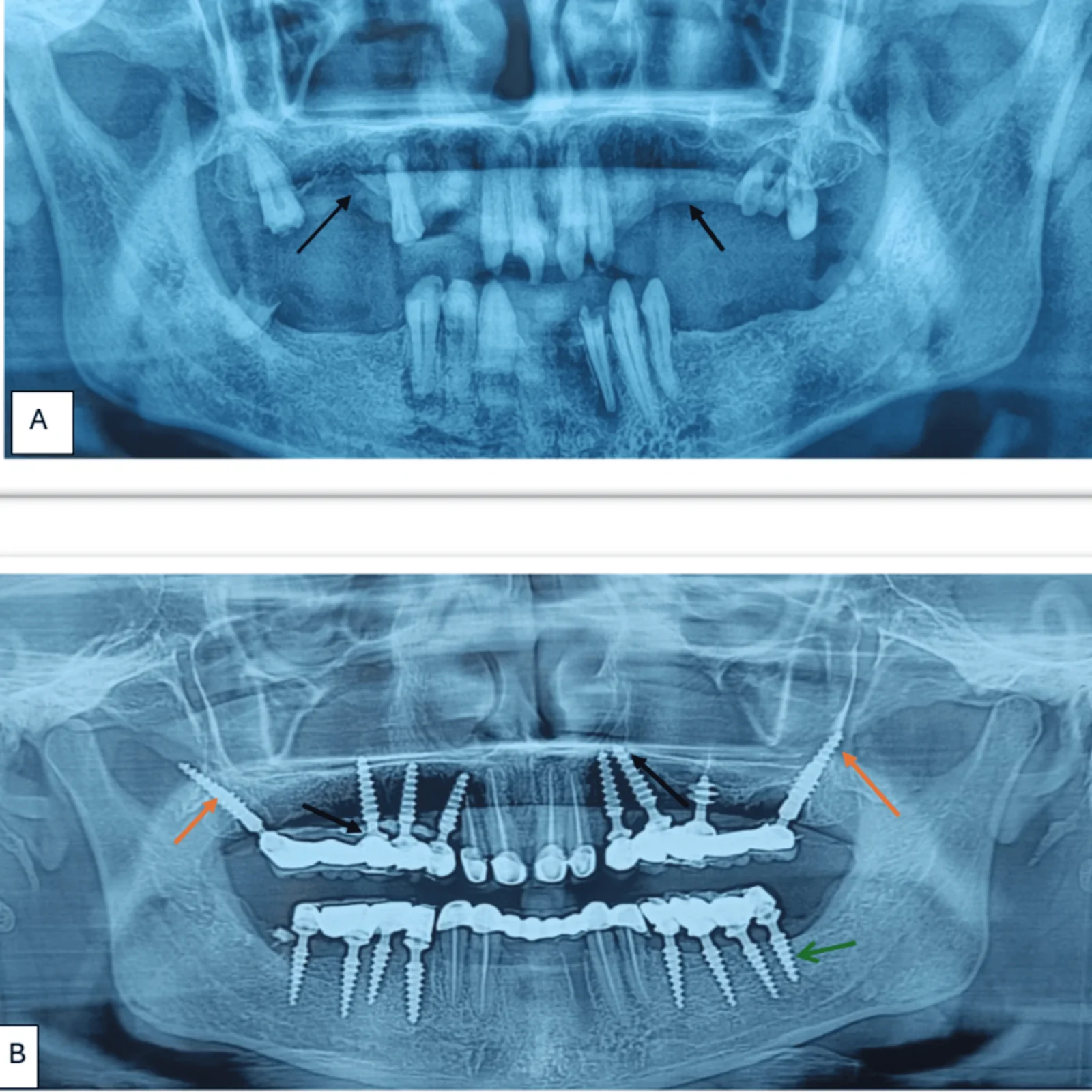
(A) Preoperative orthopantomogram showing generalized horizontal bone loss (black arrows). (B) Postoperative orthopantomogram demonstrating definitive bicortical engagement of the corticobasal implants. The orange arrows indicate strategic engagement of the pterygomaxillary region by tilted pterygoid implants, and the green arrow highlights bicortical anchorage in the mandibular basal bone.

Upon clinical examination, no mobility was noted in the remaining dentition. However, poor oral hygiene and periodontal pockets were present. Furthermore, the patient exhibited a loss of the vertical dimension of occlusion and a collapsed occlusal plane resulting from the missing posterior teeth in both arches. An orthopantomogram revealed generalized horizontal bone loss and reduced alveolar height, particularly in the posterior regions, rendering the placement of conventional implants without bone grafting unsuitable (Figure 2A).

After discussing various treatment options, the patient opted for a fixed implant-supported prosthesis using a corticobasal implant system because of its minimally invasive nature and immediate-loading capability. The finalized treatment plan involved extraction of the remaining hopeless teeth, followed by root canal treatment of teeth 11, 12, 21, 22, 34, 35, 43, 44, and 45 to serve as strategic abutments. Full-mouth rehabilitation was planned using a two-piece, multi-unit implant system. This design utilizes a separate abutment interface (multi-unit connection) rather than a single-piece implant. Immediate loading with a definitive fixed prosthesis was scheduled within 72 hours of implant placement.

Under local anesthesia, atraumatic extraction of the non-retainable teeth was performed. Corticobasal implants (Simpladent Hexacone (HC2) Implant System; Simpladent, Gommiswald, Switzerland) fabricated from Grade 4 titanium were subsequently placed. The HC2 system incorporates design elements common in modern two-piece implant systems, including an internal hex connection, a tapered interface, and a roughened surface. The implants were anchored bicortically, engaging the nasal floor, maxillary buttress, pterygomaxillary region, and mandibular basal bone to achieve primary stability greater than 70 N·cm. Postoperative radiographs confirmed ideal implant placement and cortical engagement.

The definitive fixed prostheses were fabricated and delivered within 72 hours using metal-ceramic hybrid restorations. The patient was instructed to follow a soft diet and maintain meticulous oral hygiene protocols (Figure 2B).

**Figure 3:**
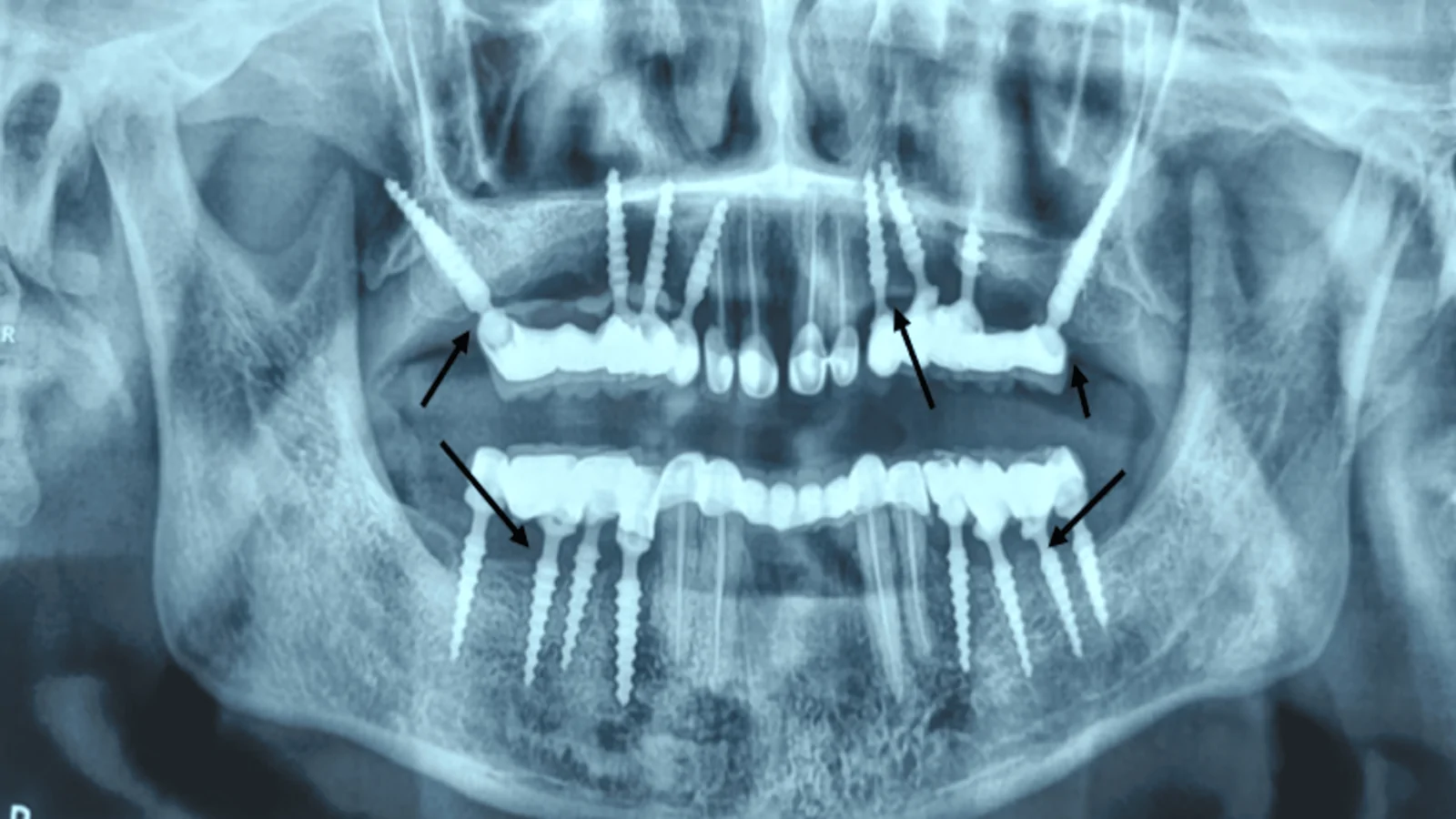
Six-month follow-up demonstrating healthy peri-implant bone (black arrows).

**Figure 4:**
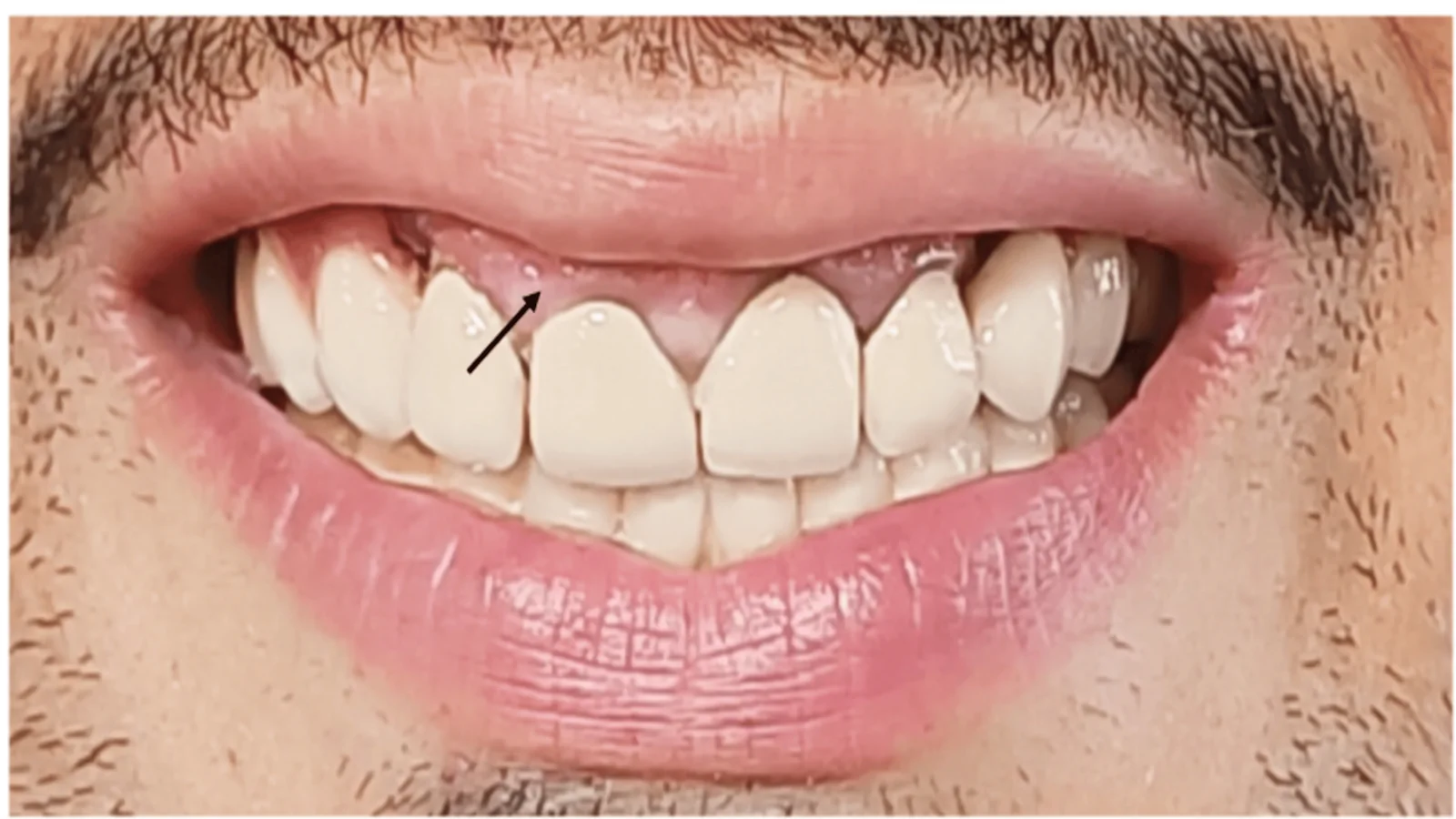
Postoperative extraoral photograph displaying the patient’s smile, demonstrating excellent soft-tissue healing (black arrow), restoration of the vertical dimension of occlusion, and an optimal aesthetic outcome.

At the three-month and six-month follow-up visits, the patient demonstrated excellent soft-tissue health, a stable occlusal relationship, and no radiographic evidence of peri-implant bone loss (Figure 3). Additionally, the patient reported marked improvement in chewing efficiency and overall satisfaction with his aesthetic appearance (Figure 4).

## Discussion

The management of patients presenting with severely compromised alveolar bone requires a strategic departure from conventional implant protocols. Traditional crestal implants rely heavily on cancellous bone, a resource that is often sparse or of poor density in patients requiring full-mouth rehabilitation. This deficiency frequently necessitates extensive, costly, and time-consuming bone grafting or sinus lift procedures. Corticobasal implantology has emerged as an alternative treatment approach for managing patients with compromised alveolar bone in whom conventional implant placement may require augmentation procedures. Unlike conventional implants, which rely primarily on cancellous bone for osseointegration, corticobasal implants engage the highly mineralized basal cortical bone. This cortical substrate exhibits lower remodeling activity and has been reported to be less susceptible to inflammatory resorption [1,2]. This biological characteristic provides high primary stability, enabling immediate functional loading even in severely atrophic ridges. Furthermore, this concept aligns with Wolff’s law, which states that bone remodels in response to functional stresses; thus, controlled mechanical loading may stimulate bone maintenance and remodeling [3,4].

In the present case, a 35-year-old male with generalized horizontal bone loss and a history of failed FPDs presented a complex restorative challenge. In addition, restoration of the lost vertical dimension of occlusion and immediate functional rehabilitation were important treatment objectives. Considering the patient’s preference for a fixed prosthesis without undergoing extensive bone grafting, a multi-unit corticobasal implant system was selected as the most appropriate treatment option. A minimally invasive strategy using Simpladent multi-unit corticobasal implants and pterygoid implants was implemented through a flapless, graftless protocol, achieving an insertion torque of >70 N·cm.

Immediate loading provided the necessary biomechanical stimulus for bone remodeling and early osseointegration. By transmitting masticatory forces directly through the dense cortical plates, functional load distribution may occur more uniformly across the basal bone, minimizing micromovements and potentially contributing to favorable early implant stability and functional rehabilitation [5,6]. This principle of rapid osseous adaptation differentiates corticobasal implantology from conventional delayed-loading protocols. Immediate loading has been reported to facilitate functional adaptation when adequate primary stability is achieved and may reduce subsequent crestal bone loss [7].

A significant advantage of this technique lies in its graftless workflow. Many patients present with extensive ridge resorption or are medically or economically unsuitable for advanced bone augmentation. In such cases, corticobasal implants may eliminate the need for sinus lifts, block grafts, or ridge-splitting procedures, thereby potentially reducing surgical morbidity, overall costs, and treatment time [8-10]. Several clinical studies have reported favorable short- and medium-term outcomes with immediately loaded corticobasal implant systems in carefully selected patients, including those with compromised bone quality [11,12]. The successful short-term outcome observed in the present patient is consistent with the findings of Lazarov, who reported high survival rates following immediate loading of corticobasal implants [5].

From a prosthodontic perspective, corticobasal implantology simplifies the rehabilitation of atrophic jaws. Immediate fixation allows for early prosthetic loading, which may improve patient comfort, phonetics, and aesthetics within 72 hours. In the present case, restoration of function and aesthetics resulted in improved chewing efficiency and patient-reported satisfaction during the six-month follow-up [13,14]. In contrast, conventional implant systems often require multiple surgical stages and prolonged edentulous periods, which may reduce patient compliance and satisfaction.

Nevertheless, careful case selection and operator experience remain crucial prerequisites for clinical success. Precise preoperative planning to ensure engagement of the second or third cortical plate, meticulous implant orientation, and rigid cross-arch splinting in full-mouth cases are essential to prevent biomechanical overload [15,16]. Long-term success also depends on appropriate occlusal adjustment and close monitoring for parafunctional habits.

The present case demonstrates that corticobasal implants can be successfully used for immediate full-mouth rehabilitation in a carefully selected patient with compromised alveolar bone. Favorable short-term functional and aesthetic outcomes were achieved without the need for bone augmentation. However, because this report describes a single patient with six months of follow-up, larger prospective studies with longer observation periods are required to establish the long-term predictability and comparative effectiveness of this treatment approach.

The present report has certain limitations. It represents the clinical outcome of a single patient with a relatively short follow-up period of six months. Objective clinical outcome measures, such as the implant stability quotient, peri-implant probing depths, plaque and bleeding indices, marginal bone level measurements, and validated patient-reported outcome measures, were not routinely recorded and therefore could not be evaluated. Future prospective studies with larger patient cohorts and longer follow-up are necessary to further validate these findings.

## Conclusions

Corticobasal implantology may provide a minimally invasive, graftless treatment option for full-mouth rehabilitation in carefully selected patients with severely resorbed alveolar ridges. By strategically utilizing dense basal cortical bone, this approach can avoid the need for extensive bone grafting or sinus augmentation procedures, potentially reducing surgical morbidity, treatment costs, and overall rehabilitation time through immediate functional loading. The favorable immediate and six-month clinical outcomes observed in this patient demonstrate the feasibility of this approach. However, as this report is limited to a single clinical case, larger prospective studies with long-term follow-up are required to validate the long-term predictability, survival rates, and stability of corticobasal implant systems before broader clinical recommendations can be established.
